# Basal axillary buds in pea are hydraulically connected to the stem but are protected during drought by osmotic adjustment

**DOI:** 10.1111/nph.70480

**Published:** 2025-08-21

**Authors:** Christopher J. Ray, Jazmine L. Humphreys, Luke A. Yates, Steven M. Smith, Timothy J. Brodribb

**Affiliations:** ^1^ School of Natural Sciences University of Tasmania Private Bag 55 Hobart TAS 7001 Australia; ^2^ Australian Research Council Centre of Excellence for Plant Success in Nature and Agriculture University of Tasmania Node Churchill Avenue Hobart TAS 7005 Australia

**Keywords:** axillary bud, drought tolerance, optical dendrometry, osmotic potential, *Pisum sativum* (garden pea), psychrometry, water potential

## Abstract

- Plants prioritise water allocation to tissues that support their survival and reproduction during drought, but little is known about their tissue‐specific drought tolerance mechanisms. In the case of basal buds, the extent to which they are hydraulically connected to the stem is unclear, as are their drought resistance mechanisms.
- To address these questions in the herbaceous annual *Pisum sativum* L. (cv. Torsdag), we used optical dendrometry to monitor the expansion and contraction of basal buds and their adjacent stems during their diurnal cycle and longer‐term during drought. We also measured water potential and osmotic potential of buds and stems.
- Dimensions of buds and stems oscillated synchronously throughout their diurnal cycle, indicating a functional hydraulic connection. Buds maintained turgor during drought by osmotic adjustment (OA) to maintain a pressure differential with the main plant xylem. Thus, immediate outgrowth of buds occurred upon rewatering.
- We conclude that OA protects buds during drought, allowing them to retain water and viability. This strategy enables growth and flowering of lateral branches postdrought, even if the primary shoot is damaged. We expect this mechanism to play a significant role in a wide range of annuals during drought, including many crop species.

## Introduction

Increasing global aridity threatens water‐availability patterns, exposing plants to more frequent and severe droughts linked to reduced crop yields (Mbow *et al*., 2019; Gupta *et al*., 2020) and forest decline (Brodribb *et al*., 2020). Plants perceive drought stress when transpiration outpaces water uptake (Bray, 1997), triggering one or more resistance strategies: Drought Escape (accelerated development), Avoidance (water retention via morphological traits), and Tolerance (osmotic and physiological adjustments) (Kooyers, 2015; Blum & Tuberosa, 2018; Martignago *et al*., 2020). These strategies can act in concert or shift with increasing stress severity (Skirycz & Inzé, 2010; Hu & Xiong, 2014), ultimately balancing survival and reproduction during water scarcity.

In woody perennials, water allocation often preserves vegetative tissues at the expense of reproductive organs to ensure future seasons of survival and reproduction (Tyree & Ewers, 1991; Bourbia *et al*., 2020; Zhang *et al*., 2020). By contrast, annuals and monocarpic perennials have only one reproductive window, making flowers and seeds the highest priority under drought (Riboni *et al*., 2016; Shavrukov *et al*., 2017; Harrison Day *et al*., 2022). As annual species account for over 80% of global food production, identifying which tissues receive preferential water allocation and understanding the physiology underpinning this priority is essential for improving agronomic practices and breeding drought‐resilient crops.

Axillary buds shape plant architecture in dicots and drive yield in many crops (Yang & Jiao, 2016; Yuan *et al*., 2024). They arise from a pluripotent cell pool at the shoot apex (Ha *et al*., 2010; Wang *et al*., 2018) and contain their own reserve of pluripotent cells but will often remain dormant until triggered to grow (Balla *et al*., 2016). Pea node‐2 buds are a well‐established model for studying this dormancy, where complex hormonal and sugar networks maintain bud arrest and rapidly initiate outgrowth following shoot apex damage (Morris, 1977; Tepper, 1993; Fichtner *et al*., 2017a,b; Barbier *et al*., 2019; Beveridge *et al*., 2023; Cao *et al*., 2023). Anatomical studies show that the formation of a vascular connection across the bud–stem junction immediately precedes bud activation (Sorokin & Thimann, 1964; Sachs, 1968), indicating that dormant buds may remain hydraulically isolated to some extent. Despite these insights, the mechanisms by which these buds regulate water uptake and turgor under soil–water deficit remain uncharacterised.

Water moves through plants along gradients of water potential (Ψ). Xylem water potential (Ψ_*X*_) follows a diurnal cycle, rising towards soil Ψ overnight and declining during daytime transpiration (van den Honert, 1948; Ehrenberger *et al*., 2012; Epron *et al*., 2021). Resistance in xylem conduits and at plant–environment interfaces shapes these Ψ_*X*_ oscillations (Dixon & Joly, 1895; Scholander *et al*., 1965; Tang & Boyer, 2002; Oberhuber *et al*., 2023; De Swaef *et al*., 2022; Affortit *et al*., 2024). Within living cells, osmotic potential (Π) and water content generate positive turgor pressure that drives cell growth and links Ψ_*X*_ oscillations to measurable tissue expansion and contraction (van't Hoff, 1887; Hsiao, 1973; Tyree & Zimmermann, 2002; Turner, 2018; Ali *et al*., 2023).

Traditional direct measures of xylem water potential (Ψ_*X*_), Scholander pressure chambers (Scholander, 1964; Tyree & Hammel, 1972) or thermocouple psychrometry, are destructive and unsuited to continuous monitoring of fragile herbaceous annuals. However, high‐resolution time‐lapse dendrometry tracks tissue expansion and contraction as a noninvasive proxy for cell turgor (McBurney, 1992; De Swaef *et al*., 2015; Dietrich *et al*., 2018; Bourbia *et al*., 2022, 2023; Bourbia & Brodribb, 2023). As turgor changes reflect Ψ_*X*_ dynamics, optical dendrometers can continuously capture tissue‐specific hydraulics, growth and behaviour during drought. When paired with periodic direct measurements of Ψ_*X*_ and Π from cohort plants, this combined approach reveals the hydraulic mechanisms that govern water‐allocation hierarchies.

As axillary bud outgrowth is critical for reproductive success after shoot damage, we hypothesise that these buds benefit from preferential water allocation, sustaining turgor under severe soil water deficit at minimal resource cost (Raven, 1985; Hummel *et al*., 2010; Blum, 2017; Munns *et al*., 2020). Potential mechanisms include increased hydraulic resistance at the bud–stem junction, osmotic adjustment (OA) within bud cells, or a combination of both. Understanding these traits can refine bud–outgrowth models and uncover new drought resistance strategies. To test our hypotheses, we compared growth, water status, and osmolarity of basal axillary buds to adjacent stem tissue in pea (*Pisum sativum*) under well watered and droughted conditions. Our results indicate a tight hydraulic connection between stem and bud, even during bud dormancy, and demonstrate OA as a key mechanism preserving bud turgor.

## Materials and Methods

### Plant material and growing conditions

Pea (*Pisum sativum* L. cv. Torsdag) seeds were sown in 2 l pots filled with densely packed, pasteurised premium potting mix (Horticultural and Landscaping Supplies, Brighton, TAS, Australia). All seed coats were nicked to standardise germination timing before sowing. Pots were arranged on two adjacent benches in a climate‐controlled glasshouse at the University of Tasmania, with weekly rotation of all plants to minimise position effects. A 12‐h photoperiod (08:30 h–20:30 h) provided natural plus supplemental lighting (*c*. 100 μmol m^−2^ s^−1^), under 40% relative humidity and 24°C : 12°C day : night temperatures. Ninety‐five plants per treatment were watered to field capacity every 2 d until day 20 postsowing. Thereafter, one cohort (*W*, *n* = 95) continued this schedule, while the droughted cohort (*D*, *n* = 95) received no water from day 20 to day 50.

Of each cohort, five plants were designated ‘sentinel’ plants for continuous optical dendrometry (see ‘Optical dendorometry’ in Materials and Methods section), and the remaining 90 ‘peripheral’ plants were sampled destructively for direct measurements of xylem water potential (Ψ) and bud/leaf osmotic potential (Π). Destructive sampling occurred predawn at six timepoints (15 plants per timepoint, *n* = 90). Final dendrometer recordings continued to day 52.

### Optical dendrometry

Optical dendrometers (cavicams.com, Hobart, TAS, Australia) recorded tissue dimensions every 5 min on sentinel plants (*W*, *n* = 5; *D*, *n* = 5) from day 15 onward. At 14 d postsowing, stipules at node 2 were excised to expose buds for imaging. Preliminary testing revealed that stipule excision induced immediate bud growth by up to 20% over a period of 18 h, so data analysis began the next day. Dendrometers were mounted to frame each node‐2 axillary bud and adjacent stem in profile, alongside a fixed black paper strip to correct for thermal expansion of camera components. Shutter speed and ISO (light sensitivity) were optimised for image sharpness per device and then kept constant; housings were covered in aluminium foil to minimise temperature and light fluctuations during imaging. Continuous bud and stem area data were extracted from time‐lapse images using imagej (see ‘Image analysis’ in Materials and Methods section).

### Image analysis

Images from optical dendrometers were analysed in imagej (https://imagej.net/ij/). For each plant, time‐lapse sequences were converted to 8‐bit greyscale and thresholded manually per sequence to preserve image sharpness and produce black and white binary images. Tissue area was quantified by counting black pixels within manually defined regions of interest (ROIs). Stem ROIs encompassed tissue near the bud‐stem junction while excluding bud structures. Bud ROIs included both bud and adjacent stem as bud‐specific area was calculated by subtracting stem ROI area from the combined ROI area (Supporting Information Fig. S1). As tissue profile areas reflect a sampling of tissue diameters within each ROI, we treated area fluctuations as a proxy for turgor dynamics (Offenthaler *et al*., 2001; Zweifel *et al*., 2005; De Schepper *et al*., 2012; Creek *et al*., 2020; Bourbia & Brodribb, 2023). A reference ROI (fixed black paper strip) corrected for thermal expansion of camera components as these reference signals were proportionally subtracted from tissue measurements. All area values were normalised to the first measurement within each time series considered for analysis.

### Xylem water potential measurement

From day 20 to day 48 at weekly intervals, and again on day 50, two fully expanded leaves were excised predawn from 15 peripheral plants per treatment (*W*, *n* = 15; *D*, *n* = 15). Predawn excision ensured equilibration of leaf and xylem Ψ (Scholander *et al*., 1965). Petioles were sealed in a Scholander pressure chamber, and pressure was gradually increased until sap exuded from the cut surface. The balancing pressure was recorded as negative xylem water potential. Peripheral plants were used to avoid defoliation effects on sentinel‐plant hydraulics and sugar signalling to axillary buds (Mason *et al*., 2014; Fichtner *et al*., 2017a,b; Barbier *et al*., 2021).

### Axillary bud osmotic potential measurements

Thermocouple psychrometers (PSY1 psychrometer, ICT International) were calibrated with standard salt solutions and used to determine osmotic potential (Π) of leaf and bud tissues. At predawn on days 20, 27, 34, 41, 48, and 50, 8 mm discs were punched from fully expanded leaves and node‐2 axillary buds were excised with a scalpel from 15 peripheral plants per treatment (*W*, *n* = 15; *D*, n = 15). Samples were flash‐frozen in liquid nitrogen, then equilibrated in a saturated‐humidity chamber for 10 min (Millar, 1974; Turner, 1981). Individual leaf discs were measured separately, whereas buds were pooled into three replicates of five buds each to ensure sufficient volume for accurate readings. Psychrometer readings yielded Π values for each leaf disc and bud pool.

### Modelling

As diurnal expansion and contraction are most pronounced at dawn and dusk, we restricted our lag analysis to ±1 h around these transitions. We quantified the temporal lag between diurnal oscillations in bud and stem relative areas using a two‐step approach. First, for droughted sentinel plants only, we fitted plant‐ and tissue‐specific generalised additive models (GAMs) to seven consecutive days of area data (days 17–23) using the R package mgcv (Wood, 2011). Each GAM included a thin‐plate smoothing spline of time with three basis functions to isolate diurnal turgor oscillations from growth trends (Oppenheim & Schafer, 2010; Wood, 2017). Second, we modelled these residuals within ±1 h of dawn and dusk transitions using nonlinear Bayesian mixed models in brms (Bürkner, 2017). We employed a logistic sigmoid function.

$$f\left(t\right)=A/\left(1+\exp\left(B*\left(t-C\right)\right)\right)+D$$

with fixed effects for *A* and *D* by plant–tissue combination (day nested as a random effect), a global intercept for *B*, and tissue‐specific fixed effects for the midpoint *C*. The difference in *C* between tissues represents the temporal lag. Four chains of 8000 iterations (2000 warmup) were run; convergence was assessed via the improved R̂ statistic (Vehtari *et al*., 2021) and models validated by posterior predictive checks (Gabry *et al*., 2019).

We then related stem area decline to xylem water potential (Ψ_*X*_, MPa) in a linear mixed effects model (lme4; Bates *et al*., 2015). The mean stem area decline (relative to prerehydration maximum) was the sole fixed effect, with measurement day as a random intercept. The dataset comprised 60 observations (15 d^−1^ on days 27, 34, 41, and 50); day 20 was excluded due to active growth. We extracted the fixed‐effect slope (95% CI via Wald profiling), computed marginal and conditional *R* ^2^, and performed a Pearson correlation (*r*, *p*).

Finally, daily rates of change in Ψ_*X*_, leaf osmotic potential (ΠL), and bud osmotic potential (ΠB) were compared across tissues and treatments using linear mixed effect models in lme4, with pairwise slope and day 27 intercept contrasts (Tukey adjustment via emmeans Lenth *et al*., 2025).

All R scripts and the datasets used for fitting the GAMs, Bayesian lag models, and mixed effect models have been deposited in a public GitHub repository (https://github.com/christopher‐ray‐publication/New‐Phytologist‐Publication‐1) to ensure full reproducibility.

## Results

### Changes in tissue dimensions during growth of well‐watered and droughted plants

Optical dendrometers were mounted to node‐2 of pea plants to record the dimensions of axillary buds and adjoining stems of well‐watered and droughted plants. The dendrometers recorded the growth of each tissue as well as oscillations in dimension due to expansion and contraction caused by turgor changes (Videos 1 and 2).

**Video 1:** Optical dendrometer time‐lapse of node‐2 bud, stem, and thermal correction reference for replicate W2 (well‐watered treatment). See Supporting Information Videos S1–, S4 for other well‐watered replicates.

**Video 2:** Optical dendrometer time‐lapse of node‐2 bud, stem, and thermal correction reference for replicate D1 (droughted treatment). Replicate label becomes blue upon rehydration. See Supporting Information Videos S5–, S8 for other droughted replicates.

**Fig. 1:**
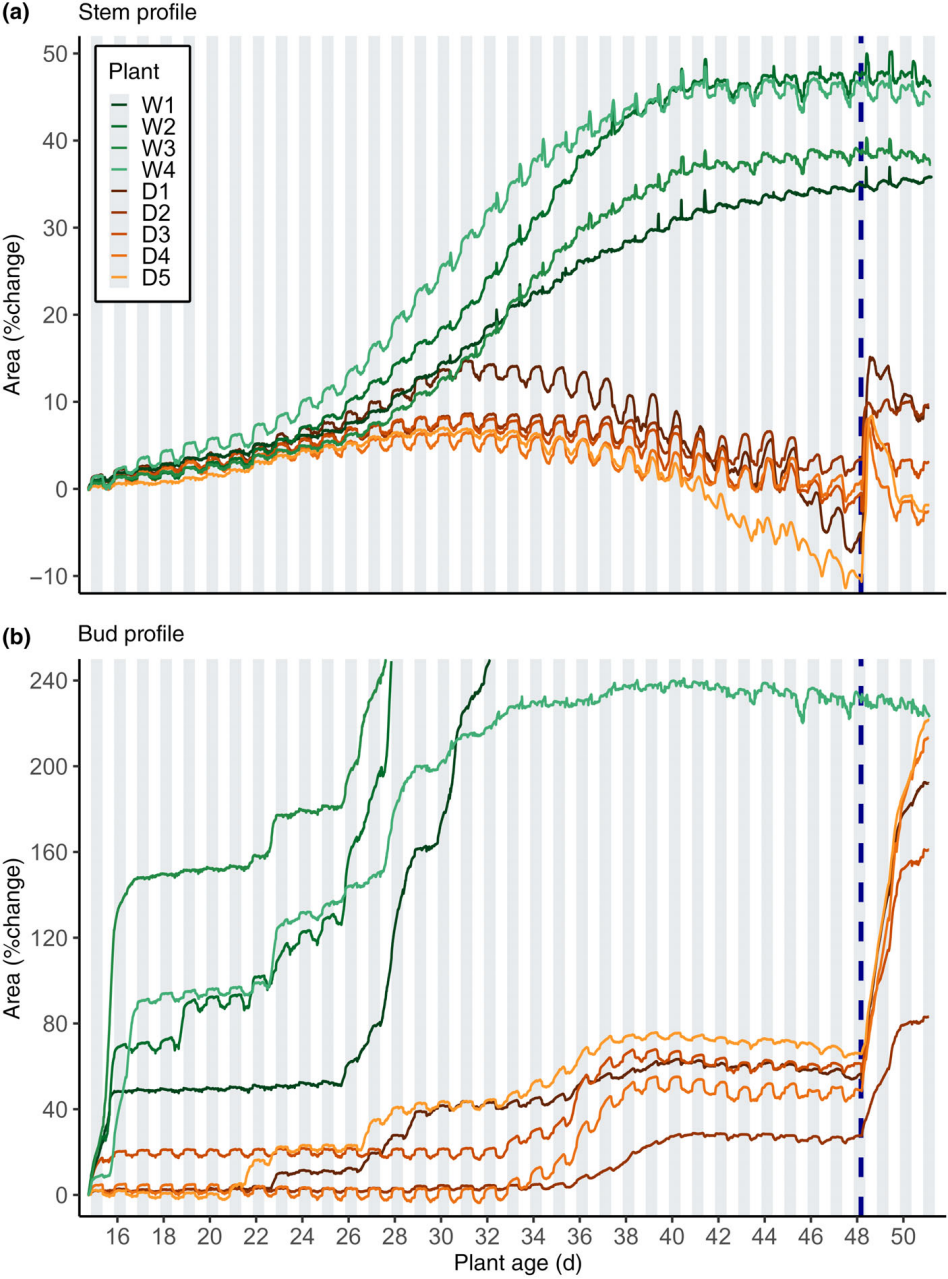
*Pisum sativum* bud and adjacent stem area relative to day 15. (a) Area change in bud‐adjacent stem profiles relative to their first measurement at plant age 15. (b) Area change in bud profiles relative to their first measurement at plant age 15. Lines denote individual plants (W1–W5, well watered; D1–D5, droughted). W5 is omitted for clarity (see Supporting Information Fig. S1). Vertical dashed blue line marks rehydration of droughted plants; shaded bars indicate darkness. W1–W3 buds grew beyond imaging frame.

A common pattern of steady growth was observed in stems of well‐watered plants until around day 44 (Fig. 1a). Buds of well‐watered plants exhibited sporadic growth until around day 28, when buds of three replicates (W1, W2, and W3) began sustained rapid outgrowth, extending beyond the frame of the dendrometers (Fig. 1b). Buds of W4 and W5 appeared arrested relative to this outgrowth after day 28.

**Fig. 2:**
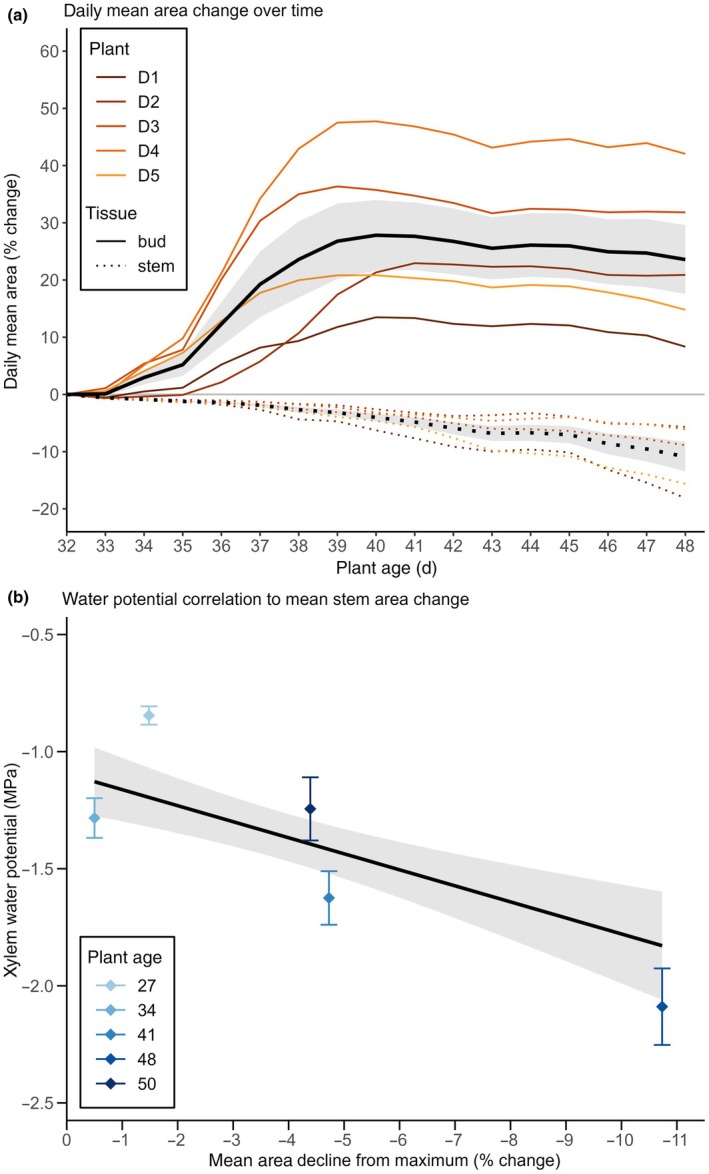
Mean tissue area in droughted plants between onset of stem area decline and rehydration compared to correlation of mean stem area decline to water potential measurements. (a) Daily mean area of *Pisum sativum* stems (dotted) and buds (solid) for droughted plants (D1–D5) from days 32–48, relative to day 32 (onset of stem area decline; Fig. 2a). Global means (black) ± SE (grey bands). (b) Relationship between mean daily stem area decline (droughted plants, *n* = 5) and xylem water potential from peripheral plants (*n* = 60). Black line: linear mixed effects fit (95% CI shaded). Pearson's *r* = −0.61; *P* < 0.001.

Stems of droughted plants exhibited a lower growth rate compared to stems of well‐watered plants, even in the first week of water deprivation, indicating high sensitivity to water deficit (Fig. 1a). These stems ceased growth around day 32 (observed tissue area maximum) and declined in overall diameter for the following 18 d (Fig. 2) but returned to maximum diameter (+0.05–2%) after rehydration on day 50 (Fig. 1a). During the period of stem diameter decline, the amplitude of the diurnal oscillations increased, which is characteristic of increasing water deficit and drought stress (Fig. 1a). In the final week before rehydration, plants began to flower, and droughted stems began to exhibit irregularities in diurnal oscillations (Fig. 1) consistent with disrupted transpiration. This was likely due to partial (or total) stomatal closure, an extreme strategy to retain water as drought lethality approaches (Ehrenberger *et al*., 2012; Bourbia *et al*., 2023; McAdam, 2023; Ziegler *et al*., 2024; Peters *et al*., 2025a,b).

We observed that stem area contractions of droughted sentinel plants coincided with decreases in xylem water potential (Ψ_*X*_) measured in peripheral plants, corroborating earlier dendrometry studies (Offenthaler *et al*., 2001; Zweifel *et al*., 2005; De Schepper *et al*., 2012; Creek *et al*., 2020; Bourbia & Brodribb, 2023; Peters *et al*., 2025a,b). A linear mixed effect model with random intercepts for measurement day showed that each 1% reduction in stem area predicted a 0.102 MPa drop in Ψ_*X*_ (95% CI: 0.041–0.163, *P* < 0.001). The fixed effects alone explained 36.8% of the variance (marginal *R* ^2^ = 0.368), rising to 47.1% when daily intercepts were included (conditional *R* ^2^ = 0.471). A simple Pearson correlation on the raw data (*r* = −0.607, *p* ≤ 0.001) further confirms a strong, negative association between stem contraction and water potential.

Previous within‐plant calibrations of optical dendrometry against simultaneous pressure chamber Ψ_X_ measurements have yielded even higher fits (*R* ^2^ = 0.92–0.99; Bourbia & Brodribb, 2023). However, repeated pressure chamber sampling on the same individual can remove a substantial portion of leaf area, an intervention that disturbs water status and growth. To avoid this artefact, we instead calibrated sentinel stem area contraction against Ψ_*X*_ of peripheral plants, leveraging natural between‐plant variation to capture population‐level hydraulic dynamics. The high within‐plant correlations previously reported lend confidence to our peripheral–plant calibration for estimating sentinel Ψ_*X*_.

Before rehydration, buds of droughted plants exhibited sporadic bursts of growth, although they were of smaller magnitude and occurred later than in well‐watered plants (Fig. 2b). These bursts of bud growth occurred when their adjacent stems were contracting (Figs 1, 2). Quantitation of relative growth of stems and buds in droughted plants shows that stems grew until around day 30, whereas bud growth continued for at least another 10 d (Fig. 1). Rehydration of droughted plants triggered immediate bud growth, whereas stems of droughted plants rapidly lost water after transient rehydration (Fig. 1).

Remarkably, droughted buds consistently displayed resilience to sustained systemic water deficit and were able to maintain mean daily area and diurnal area oscillation amplitude even as their adjacent stems contracted, indicating a tissue‐specific drought tolerance that prioritises bud water status and permits their rapid growth when water availability resumes (Figs 1, 2a).

### Synchronisation of diurnal oscillations of stems and buds

**Fig. 3:**
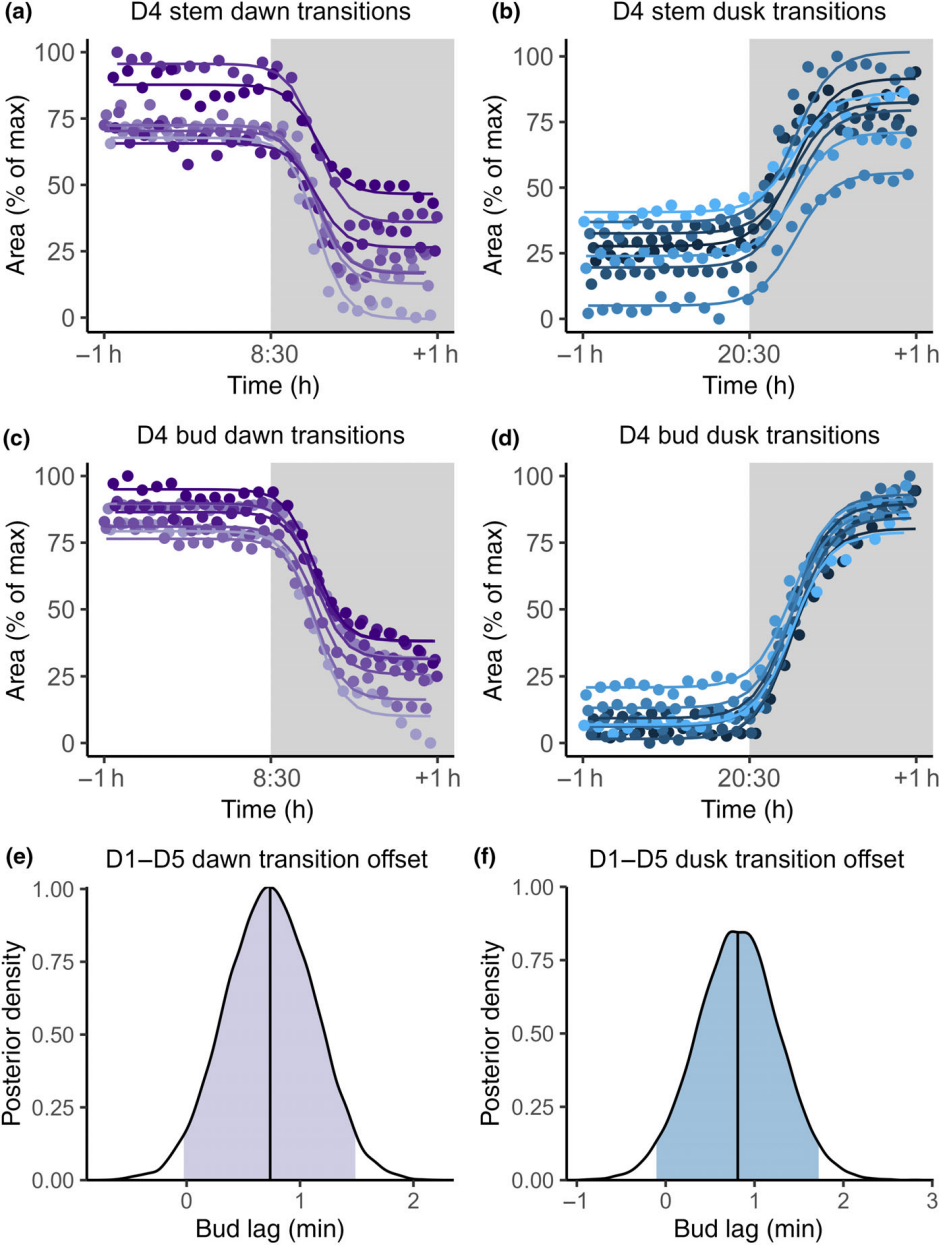
Bayesian lag modelling of *Pisum sativum* (D4) tissue responses during light transitions (plant ages 17–24). (a–d) Observed data points expressed as a percentage of the maximum during the days considered and fitted sigmoidal curves for each day; colours indicate same‐day data. Shading denotes darkness. Data span ±1 h around each dawn and dusk. See Supporting Information Figs S2–S5 for other replicates. (e, f) Posterior densities of the estimated lag in droughted plants (D1–D5), with vertical lines at the mean and shaded 95% CI.

A key factor in establishing the extent of hydraulic connectivity between stems and buds is to determine if there is any lag between the diurnal oscillations of each tissue. We therefore modelled the rapid changes in tissue areas in droughted plants (D1**–**D5) during 1 wk of dawn and dusk transitions (Fig. 3a–d). Data were modelled using a sigmoidal curve, and the timing of midpoint inflection was determined for both bud and stem. Typically, the tissue area changed over a period of *c*. 30 min, but the expected difference in the midpoint inflection times between stem and bud differed by < 1 min for each transition type, with each 95% CI also encompassing 0 (Fig. 3e,f). These results indicate negligible lag between stem and bud tissue and therefore a strong hydraulic connection between them.

### Xylem water potential compared to osmotic potential of buds

**Fig. 4:**
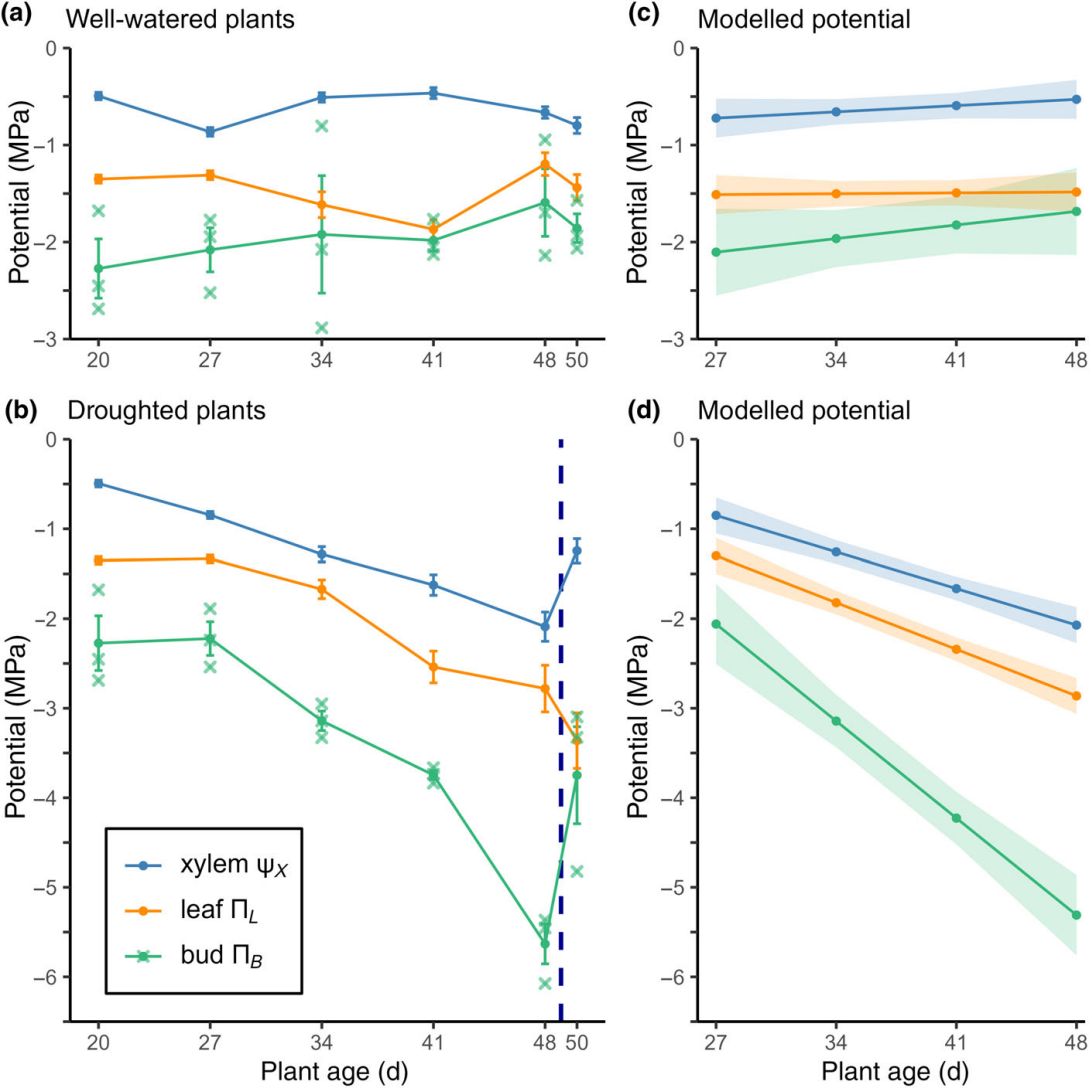
Observed and modelled tissue‐specific water status dynamics in well‐watered and droughted *Pisum sativum*. (a, b) Time courses of mean xylem water potential (Ψ_*X*_), leaf osmotic potential (Π_*L*_), and bud osmotic potential (Π_*B*_) under well‐watered and droughted regimes. Measurements taken from new peripheral plants at each timepoint (*n* = 15): Ψ_*X*_ and Π_*L*_ from individuals and Π_*B*_ from pools of five buds (X‐marks). Error bars represent ± SE. Dashed blue line, rehydration. (c, d) Modelled trajectories for Ψ_*X*_, Π_*L*_, and Π_*B*_ as functions of plant age. Solid lines, predicted means; shaded bands, 95% CI.

Mean Ψ_*X*_ of well‐watered plants remained within a narrow range between −0.47 and −0.87 MPa, while droughted plants exhibited a steady decline in mean Ψ_*X*_ as drought severity increased, reaching a low of −2.09 MPa (Fig. 4). Upon rehydration, droughted plant Ψ_*X*_ recovered but did not reach previous highs despite stem areas briefly returning to the recorded maximum, although Ψ_*X*_ measurements were taken on day 50 when stem area had already declined (Fig. 1a). Mean bud osmotic potential (Π_*B*_) exhibited similar dynamics to Ψ_*X*_ in both conditions, maintaining a consistent pressure differential of at least 0.92 MPa (Fig. 4). In the case of severely droughted plants (day 48), mean Π_*B*_ reached a remarkable −5.63 MPa to achieve a pressure differential to Ψ_*X*_ of 3.54 MPa (Fig. 4b). The modelled droughted Π_*B*_ slope and day 27 intercepts differed significantly from those of droughted Ψ_*X*_ (*P* < 0.001), demonstrating significantly different baselines and a widening pressure differential as drought stress increased (Fig. 4d). Mean leaf osmotic potential (Π_*L*_) appeared more variable but remained close to mean Ψ_*X*_, exhibiting a smaller pressure differential to Ψ_*X*_ than buds (Fig. 4a,b). Modelled day 27 intercepts of Π_*L*_ and Ψ_*X*_ pairs in both conditions were significantly different (*P* < 0.05), while their slopes did not differ significantly within each condition. Mean Π_*L*_ did not exhibit any recovery coincident with the rehydration of droughted plants. Combined, these findings show that Π_*L*_ declines at the same rate as Ψ_*X*_ as drought stress increases, but that dynamic OA and recovery do not occur in leaves to the same extent as in buds (Fig. 4b). These results suggest active OA of bud tissue to resist water loss to neighbouring tissue and that buds are positioned higher on the water allocation hierarchy than other vegetative tissue such as stems and leaves.

## Discussion

### Tissue‐specific drought tolerance

We demonstrate that node‐2 axillary buds maintain turgor throughout severe soil water deficit, even as adjacent stems lost turgor. These findings indicate a tissue‐specific drought tolerance mechanism that prioritises water allocation to axillary buds over other vegetative tissues and emphasises their importance in the pea survival strategy. We observed that axillary bud area, as a proxy for turgor (Offenthaler *et al*., 2001; Zweifel *et al*., 2005; De Schepper *et al*., 2012; Creek *et al*., 2020; Bourbia & Brodribb, 2023), does not decrease through severe water deficit while adjacent stem tissue contracts by up to 18%. Bud turgor maintenance continued as diurnal oscillations in stem turgor destabilised, typical of stomatal closure, an extreme strategy employed under critical drought stress that trades photosynthetic efficiency for water retention. Previously characterised vulnerability curves for *P. sativum* show a catastrophic loss of hydraulic conductivity in the region of −1.7 MPa xylem water potential (Ψ_*X*_) (Harrison Day *et al*., 2024); whereas we observed a mean low of −2.09 MPa Ψ_*X*_.

Therefore, our findings indicate that basal axillary buds are robust to the Ψ_*X*_ range that pea encounters as it approaches lethal drought stress. Analysis of synchrony between diurnal bud and stem turgor oscillations found negligible temporal lag, suggesting hydraulic resistance is insufficient to explain bud turgor maintenance. Direct measurements of Ψ_*X*_ and bud osmotic potential (Π_*B*_) revealed the maintenance of *a* > 0.9 MPa pressure differential as both declined under continued water deficit. Together, these results support a model in which basal buds actively lower their osmotic potential below that of adjacent stems, retaining water throughout severe soil drying and safeguarding the buds essential for postdrought recovery.

### Drought resistance strategy includes bud preservation

Reproductive success, tested against countless instances of drought across evolutionary history, directs the evolution of drought resistance. Therefore, we expect the different reproductive strategies of annuals and perennials to correspond to drought resistance mechanisms that best support these strategies. Perennials tend to preserve vegetative tissue over reproductive tissue in times of abiotic stress, supporting their strategy of surviving to subsequent years with more favourable conditions (Tyree & Ewers, 1991; Bourbia *et al*., 2020; Zhang *et al*., 2020; Carins‐Murphy *et al*., 2023). Herbaceous annuals tend to prioritise water supply to reproductive tissues during drought, supporting their strategy of maximising reproductive success within a single growing season (Harrison Day *et al*., 2022; Harrison Day & Brodribb, 2023). It is then surprising that in *P. sativum*, we found that water allocation to vegetative node‐2 axillary buds was maintained throughout severe water deficit and even during flowering (as had begun by day 48). This apparent contradiction may be explained by the well‐established role of node‐2 axillary buds as a rapidly deployed replacement of the shoot apex after its destruction (Ha *et al*., 2010; Rameau *et al*., 2015). Shoot apex destruction diminishes reproductive capacity. Axillary buds, and especially the ‘first‐responder’ node‐2 axillary buds, are therefore a special case of vegetative tissue containing the pluripotent stem cells required to mitigate losses in reproductive capacity. It is then practical that a drought resistance strategy in pea should include the preservation of these buds at minimal resource cost. However, *P. sativum* roots are not significantly more resistant than aerial tissue to cavitation, a symptom of drought stress in which embolisms may permanently obstruct xylem and isolate sections of aerial tissue from water supply (Rodriguez‐Dominguez *et al*., 2018; Harrison Day *et al*., 2024). It is then only the long intervening xylem (and the commensurate Ψ gradient) between the roots and apex that tips the balance towards apex isolation by cavitation occurring before basal buds (Cardoso *et al*., 2022; Rodríguez‐Domínguez *et al*., 2023). Indeed, the likelihood of embolism obstruction to aerial tissue increasing with xylem distance from roots may explain the capacity for observed stems situated at the plant's base to rehydrate to previous maximums after reaching a mean Ψ_*X*_ low of −2.09 MPa, far lower than previously defined p50s for *P. sativum* (−1.7 MPa; Harrison Day *et al*., 2024). It is possible that the proximity of basal buds and stem segments to the roots extends their access to water, permitting resprouting as a form of recovery from otherwise lethal drought.

### Stem to bud hydraulic connectivity

Hydraulic resistance between axillary buds and adjacent stems *R*_bud–stem_ was one mechanism considered to explain bud turgor maintenance. Anatomical (Sorokin & Thimann, 1964; Sachs, 1968) and molecular studies (Brewer *et al.*, 2015; Chabikwa *et al*., 2019; Nahas *et al*., 2024) of axillary buds indicate that vasculature development immediately precedes their outgrowth, suggesting that dormant buds may be hydraulically isolated to some extent. Furthermore, there are examples of xylem conductance and symplastic permeability adjustment in other bud types across species (Rinne & van der Schoot, 1998; Viola *et al*., 2007; Lee *et al*., 2017; Yang *et al*., 2020). To address our specific research question, we assumed that *R*_bud–stem_, to an extent necessary for buds to resist water loss, would necessarily result in a time lag between the diurnal oscillations of these tissues. Diurnal oscillations in tissue area are determined by water inflow through roots and outflow through the canopy during transpiration (Zweifel *et al*., 2005; Bourbia & Brodribb, 2023). Therefore, significant hydraulic resistance between axillary buds and the main vasculature would desynchronise their diurnal oscillation signals (Milne *et al*., 1983). Analysis of optical dendrometry data found negligible lag in dormant buds (Fig. 3). We therefore conclude that these buds maintain strong hydraulic connectivity and must therefore approach the water potential of the xylem, indicating observed differences in turgor are likely due to osmoregulation.

### Osmotic adjustment of buds

With a robust hydraulic connection established, Π_*B*_ adjustment remained as a likely mechanism for turgor maintenance, assuming predawn values are not significantly adjusted after dawn. Axillary bud cell turgor (P_*t*_) is determined by the pressure difference between Ψ_*X*_ and Π_*B*_:

$$P_{t}=\Psi_{X}–\Pi_{B}$$

We observed that as Ψ_*X*_ declined through drought and recovered after rehydration, Π_*B*_ followed at lower pressure, maintaining a pressure difference > 0.92 MPa. Trajectory modelling revealed a significant difference between Ψ_*X*_ and Π_*B*_ baselines and slopes at the onset of drought stress (*P* > 0.001 for each), showing that the pressure differential increases with drought severity. While the minimal requirement for water retention is the steady maintenance of a pressure differential, an increasing differential offers a buffer against sudden drops in Ψ_*X*_. This indicates that axillary buds adjust their Π_*B*_ to stabilise turgor pressure to *c*. 1 MPa or more as Ψ_*X*_ fluctuates according to water availability. Mean Π_*B*_ was measured at −2.27 MPa before drought and is then adjusted to a mean low of −5.63 MPa before rehydration, maintaining high P_*t*_ and far exceeding OA observed across all crop species studied barring rice (Blum, 2017; Chimenti *et al*., 2006; Morgan, 1977; Sánchez *et al*., 1998; Subbarao *et al*., 2000; Turner, 2018). These findings are therefore sufficient to account for the observed maintenance of axillary bud tissue turgor throughout severe water deficit stress and even during early flowering, emphasising their importance as a possible pathway to postdrought recovery. Given the node‐2 bud sensitivity to sugars as a trigger for outgrowth (Mason *et al*., 2014; Fichtner *et al*., 2017a,b), OA is likely facilitated by other osmolytes either synthesised locally or delivered against the water pressure gradient via selective channels (Zhu, 2002; Martinoia *et al*., 2007; Szabados & Savouré, 2010; Sevilem *et al*., 2013). Furthermore, OA for axillary bud turgor maintenance is a probable mechanism given their role as a rapidly deployed replacement of the shoot apex. Not only is high turgor pressure essential for rapid cell division and therefore bud outgrowth (Dumais, 2021; Ali *et al*., 2023), but its maintenance by OA is likely to impose a trivial resource cost (Raven, 1985; Hummel *et al*., 2010; Blum, 2017; Munns *et al*., 2020).

### Conclusion

Demonstrating node‐2 axillary bud preservation through severe water deficit in *P. sativum* provides novel insights into tissue‐specific drought resistance and how these support annual reproductive strategies. However, tissue‐specific drought tolerance and the mechanisms underpinning these adaptive traits have yet to be fully explored, particularly in herbaceous annuals where water status is difficult to evaluate. As most food crops are herbaceous annuals, studying their drought response mechanisms is crucial to inform vulnerability estimates and growing practices as climate change increases aridity and destabilises water availability. Our results show that node‐2 axillary buds adjust their osmotic potential to maintain turgor pressure, preserving their ability for rapid growth when required. Further study of the molecular mechanisms regulating osmotic potential, including identification of the specific osmolytes involved, holds significant potential for enhancing drought resilience.

## Competing interests

None declared.

## Author Contributions

CJR, TJB, SMS, and JLH designed the research. CJR performed experiments. CJR and LAY analysed data. CJR, TJB, LAY, JLH, and SMS interpreted the data. CJR, LAY, and SMS wrote the manuscript. CJR, TJB, SMS, LAY, and JLH edited the manuscript.

## Disclaimer

The New Phytologist Foundation remains neutral with regard to jurisdictional claims in maps and in any institutional affiliations.

## Supporting information


**Fig. S1** Area (% change) of bud and stem profiles including all replicates.
**Fig. S2** Bayesian regression modelling of dawn and dusk transitions (plant D1).
**Fig. S3** Bayesian regression modelling of dawn and dusk transitions (plant D2).
**Fig. S4** Bayesian regression modelling of dawn and dusk transitions (plant D3).
**Fig. S5** Bayesian regression modelling of dawn and dusk transitions (plant D5).
**Methods S1** Staging diagram of reference, stem, and bud for dendrometry.

**Video S1** Timelapse of W1 node‐2 bud and adjacent stem.

**Video S2** Timelapse of W3 node‐2 bud and adjacent stem.

**Video S3** Timelapse of W4 node‐2 bud and adjacent stem.

**Video S4** Timelapse of W5 node‐2 bud and adjacent stem.

**Video S5** Timelapse of D2 node‐2 bud and adjacent stem.

**Video S6** Timelapse of D3 node‐2 bud and adjacent stem.

**Video S7** Timelapse of D4 node‐2 bud and adjacent stem.

**Video S8** Timelapse of D5 node‐2 bud and adjacent stem.
Please note: Wiley is not responsible for the content or functionality of any Supporting Information supplied by the authors. Any queries (other than missing material) should be directed to the *New Phytologist* Central Office.

## Data Availability

The data that support the findings of this study are available in the Supporting Information of this article and in the GitHub repository found at https://github.com/christopher‐ray‐publication/New‐Phytologist‐Publication‐1.
