# Supplementary material for: Basal axillary buds in pea are hydraulically connected to the stem but are protected during drought by osmotic adjustment
Source: New Phytol. 2025 Aug 21;248(4):1700–12. doi: 10.1111/nph.70480 (PMC13020658; doi:10.1111/nph.70480)
Supplement: Supplementary file 3 — Fig. S1 Area (% change) of bud and stem profiles including all replicates. Fig. S2 Bayesian regression modelling of dawn and dusk transitions (plant D1). Fig. S3 Bayesian regression modelling of dawn and dusk transitions (plant D2). Fig. S4 Bayesian regression modelling of dawn and dusk transitions (plant D3). Fig. S5 Bayesian regression modelling of dawn and dusk transitions (plant D5). Methods S1 Staging diagram of reference, stem, and bud for dendrometry. [file NPH-248-1700-s001.docx]

## *New Phytologist* Supporting Information

Article title: Basal axillary buds in pea are hydraulically connected to the stem but are protected during drought by osmotic adjustment.

Authors: Christopher J. Ray, Jazmine L. Humphreys, Luke A. Yates, Steven M. Smith and Timothy J. Brodribb.

Article acceptance date: 15 July 2025

The following Supporting Information is available for this article:

**Figure S1** Area (%change) of bud and stem profiles including all replicates.

**Figure S2** Bayesian regression modelling of dawn and dusk transitions (plant D1).

**Figure S3** Bayesian regression modelling of dawn and dusk transitions (plant D2).

**Figure S4** Bayesian regression modelling of dawn and dusk transitions (plant D3).

**Figure S5** Bayesian regression modelling of dawn and dusk transitions (plant D5).

**Method S1** Staging diagram of reference, stem, and bud for dendrometry.

**Video S1** Timelapse of W1 node-2 bud and adjacent stem.

**Video S2** Timelapse of W3 node-2 bud and adjacent stem.

**Video S3** Timelapse of W4 node-2 bud and adjacent stem.

**Video S4** Timelapse of W5 node-2 bud and adjacent stem.

**Video S5** Timelapse of D2 node-2 bud and adjacent stem.

**Video S6** Timelapse of D3 node-2 bud and adjacent stem.

**Video S7** Timelapse of D4 node-2 bud and adjacent stem.

**Video S8** Timelapse of D5 node-2 bud and adjacent stem.

**
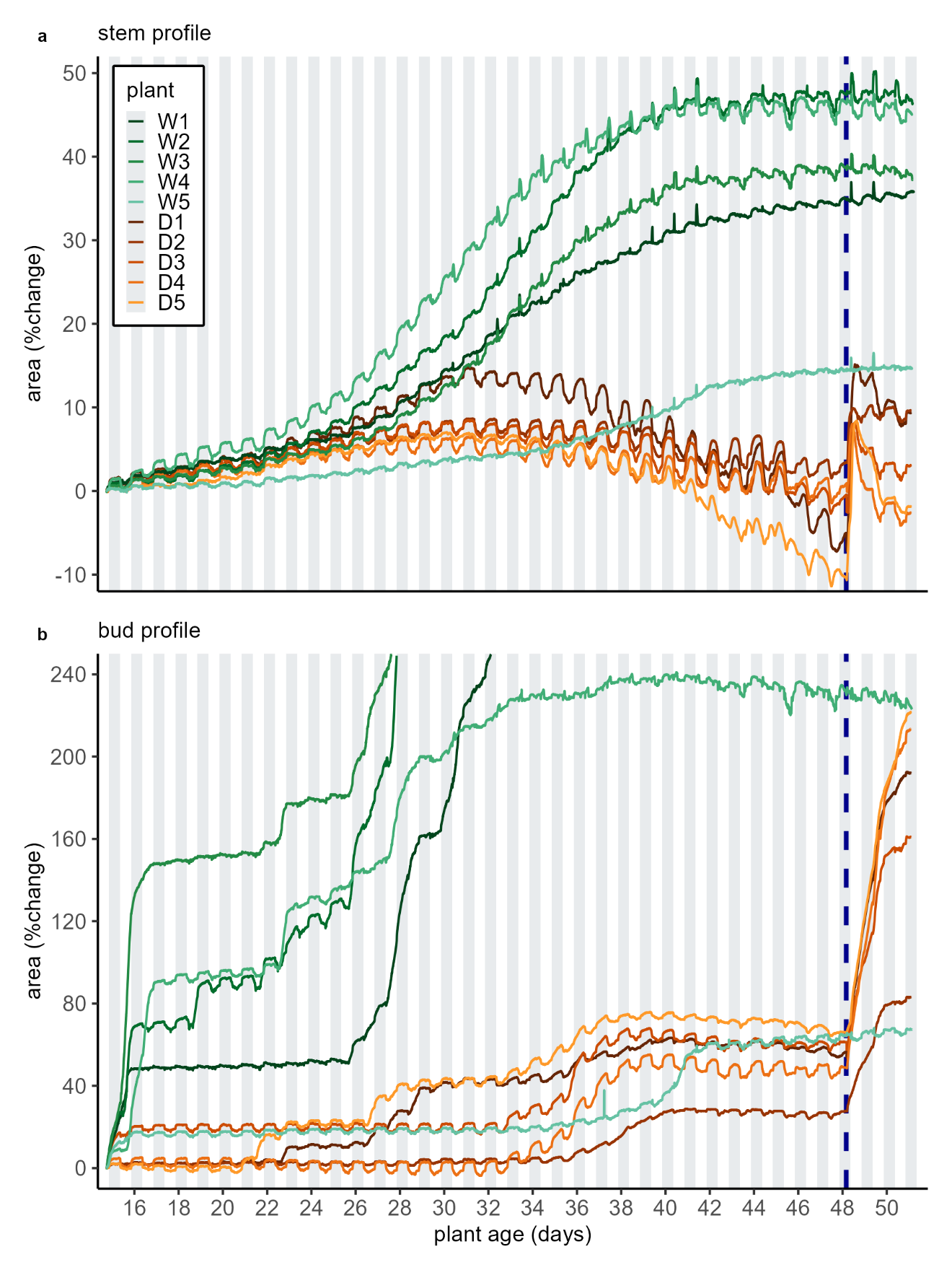
**

**Figure S1.** *Pisum sativum* bud and adjacent stem area relative to day 15. Lines denote individual plants (W1–W5 = well-watered; D1–D5 = droughted). Vertical dashed blue line marks rehydration of droughted plants; shaded bars indicate darkness. W1–W3 buds grew beyond imaging frame.

**
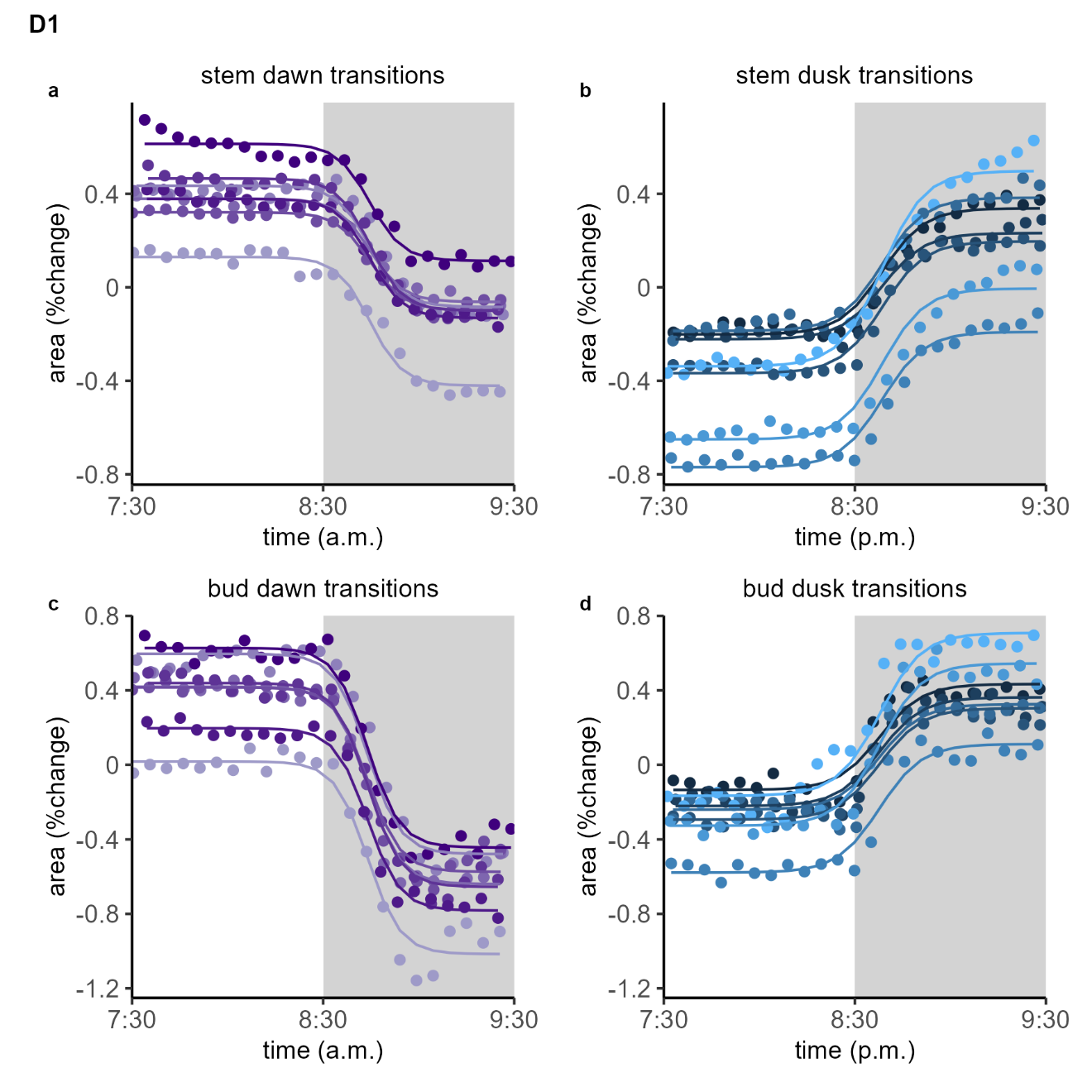
**

**Figure S2.** Bayesian lag modelling of *Pisum sativum* (D1) tissue responses during light transitions (days 17–24). **a–d:** Observed data points expressed as percentage of the maximum during days considered and fitted sigmoidal curves for each day; colours indicate same-day data. Grey shading denotes darkness. Data span ±1 h around each dawn and dusk.


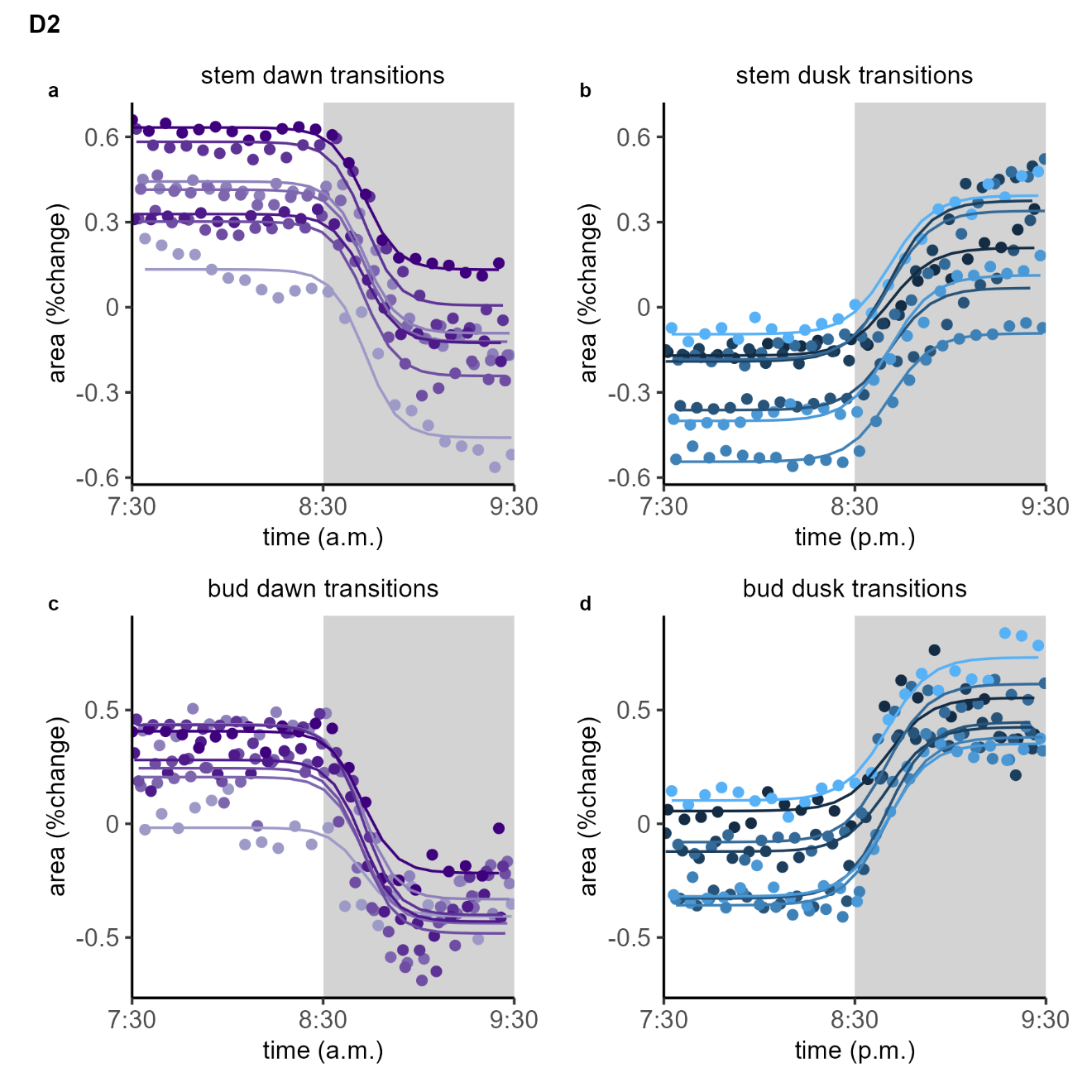


**Figure S3.** Bayesian lag modelling of *Pisum sativum* (D2) tissue responses during light transitions (days 17–24). **a–d:** Observed data points expressed as percentage of the maximum during days considered and fitted sigmoidal curves for each day; colours indicate same-day data. Grey shading denotes darkness. Data span ±1 h around each dawn and dusk.

**
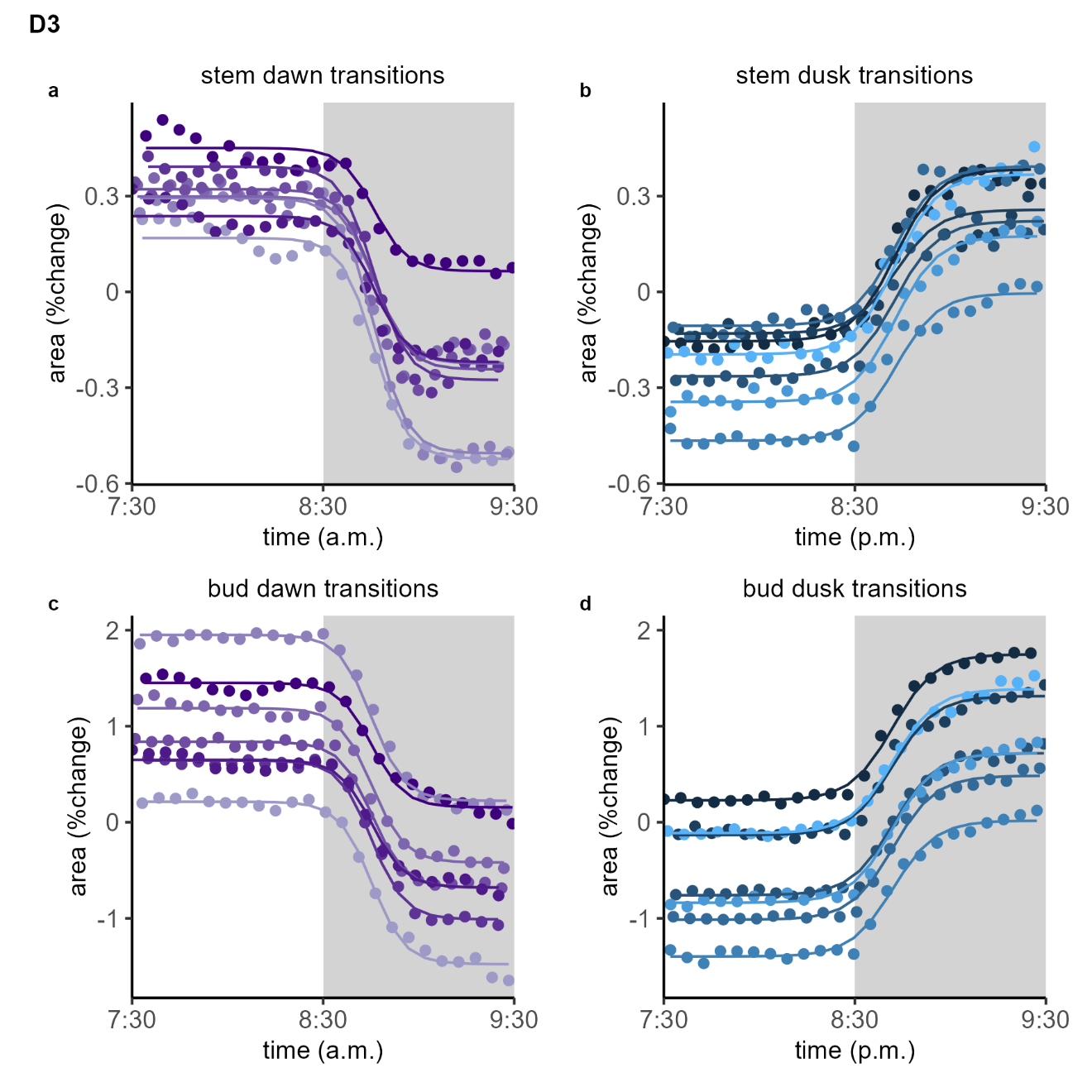
**

**Figure S4.** Bayesian lag modelling of *Pisum sativum* (D3) tissue responses during light transitions (days 17–24). **a–d:** Observed data points expressed as percentage of the maximum during days considered and fitted sigmoidal curves for each day; colours indicate same-day data. Grey shading denotes darkness. Data span ±1 h around each dawn and dusk.

**
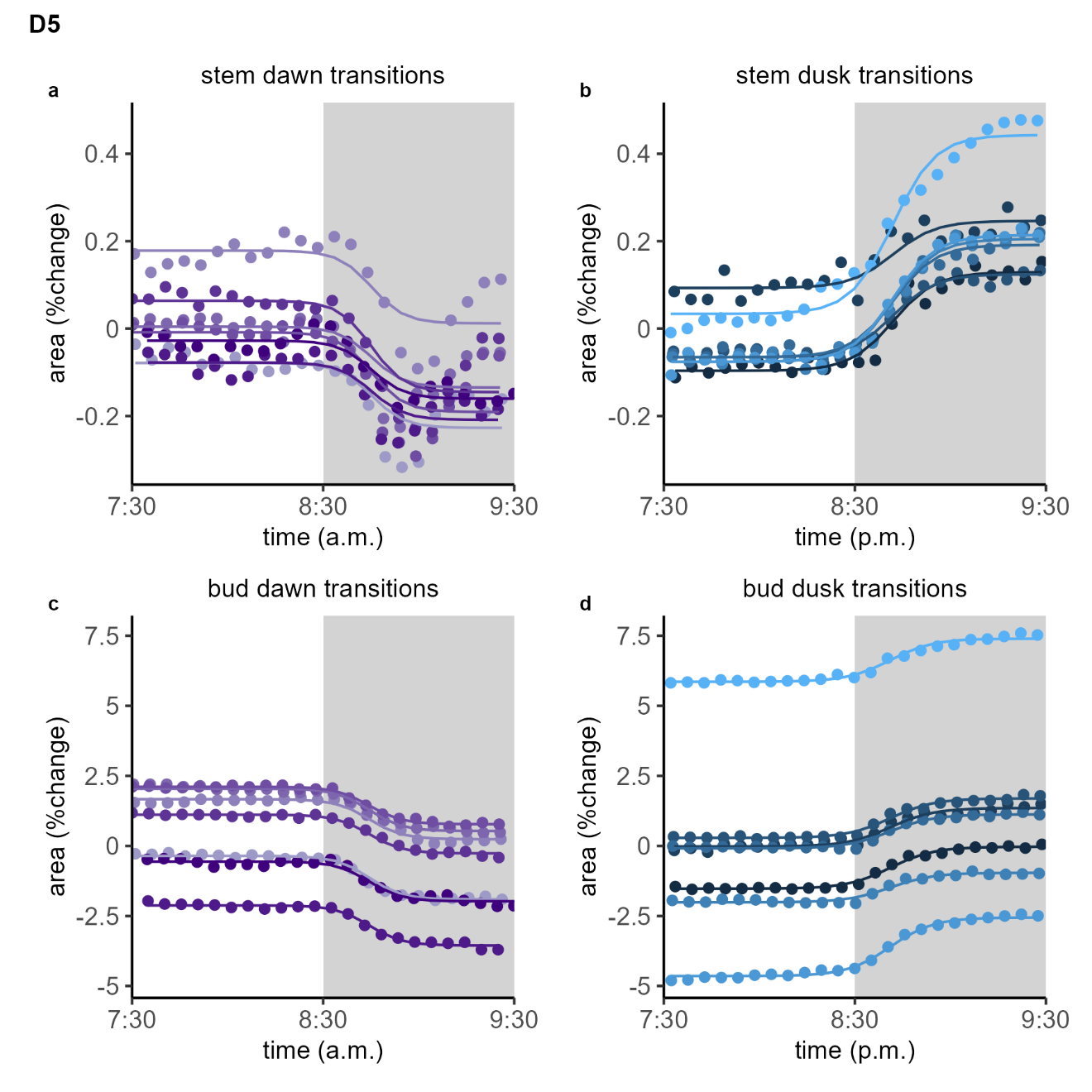
**

**Figure S5.** Bayesian lag modelling of *Pisum sativum* (D5) tissue responses during light transitions (days 17–24). **a–d:** Observed data points expressed as percentage of the maximum during days considered and fitted sigmoidal curves for each day; colours indicate same-day data. Grey shading denotes darkness. Data span ±1 h around each dawn and dusk.

**
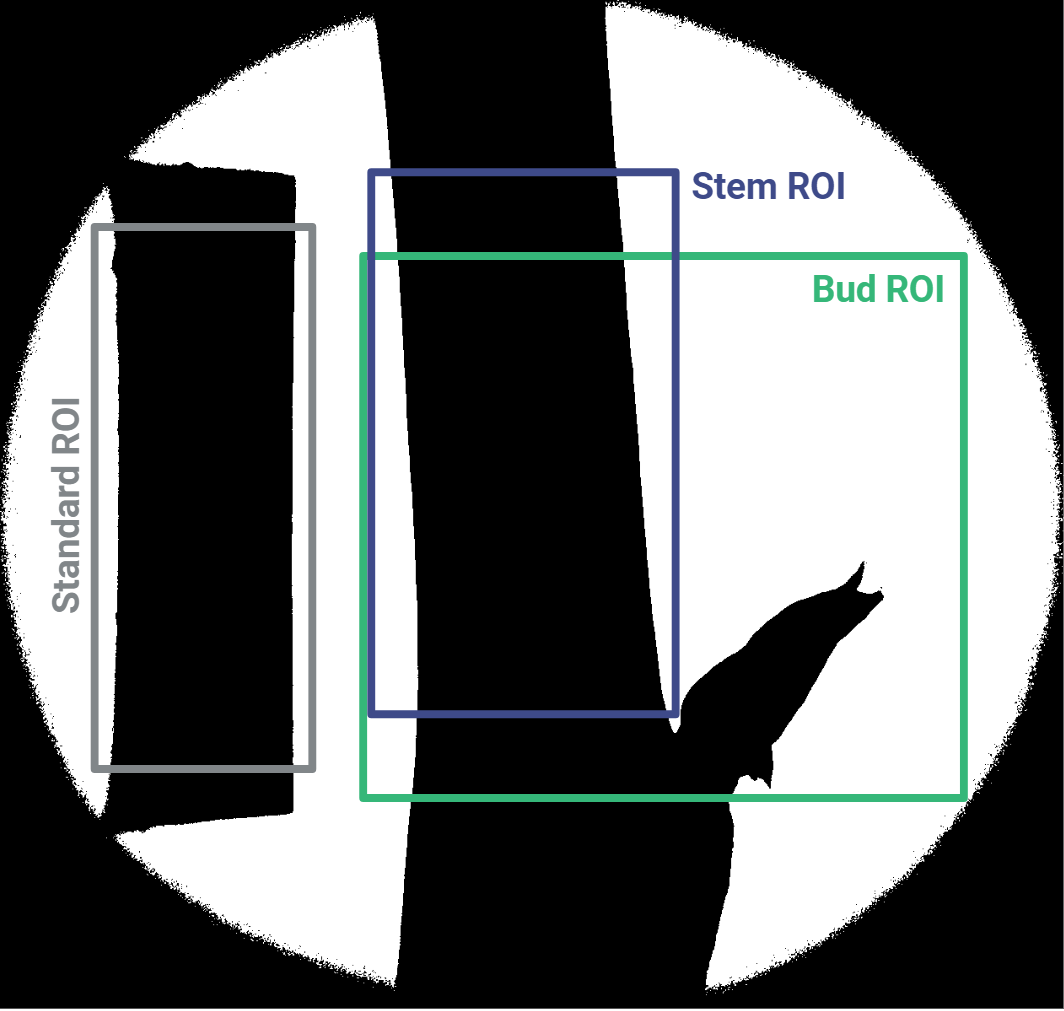
**

**Methods S1.** Example of optical dendrometry image converted to binary with stem, bud and reference area region of interest (ROI) window positions. Stems were measured directly from stem ROI. Bud measurements were calculated by subtracting stem measurements from bud ROI measurements. Proportional thermal expansion acting on the dendrometer was calculated from fluctuations in reference area ROI and subtracted from stem and isolated bud measurements.

**Video S1.** Optical dendrometer time-lapse of node-2 bud, stem and thermal correction reference for replicate W1 (well-watered treatment).

**Video S2.** Optical dendrometer time-lapse of node-2 bud, stem and thermal correction reference for replicate W3 (well-watered treatment).

**Video S3.** Optical dendrometer time-lapse of node-2 bud, stem and thermal correction reference for replicate W4 (well-watered treatment).

**Video S4.** Optical dendrometer time-lapse of node-2 bud, stem and thermal correction reference for replicate W5 (well-watered treatment).

**Video S5.** Optical dendrometer time-lapse of node-2 bud, stem and thermal correction reference for replicate D2 (droughted treatment). Replicate label becomes blue upon rehydration.

**Video S6.** Optical dendrometer time-lapse of node-2 bud, stem and thermal correction reference for replicate D3 (droughted treatment). Replicate label becomes blue upon rehydration.

**Video S7.** Optical dendrometer time-lapse of node-2 bud, stem and thermal correction reference for replicate D4 (droughted treatment). Replicate label becomes blue upon rehydration.

**Video S8.** Optical dendrometer time-lapse of node-2 bud, stem and thermal correction reference for replicate D5 (droughted treatment). Replicate label becomes blue upon rehydration.
